# Validation of pharmacokinetic model for quizartinib quantified by UPLC-MS/MS in patients with FLT3-ITD negative newly diagnosed acute myeloid leukemia

**DOI:** 10.1007/s00228-025-03909-4

**Published:** 2025-08-30

**Authors:** Antonio Solana-Altabella, Carles Iniesta-Navalón, Maria Chovi-Trull, Rebeca Rodriguez-Veiga, Marina Lopez-Nogueroles, David Martínez-Cuadrón, Mayte Gil-Candel, Laura Torres-Miñana, Evelyn Acuña-Cruz, Isabel Cano-Ferri, Blanca Boluda, Irene Navarro-Vicente, Pilar Lloret-Madrid, Eva Barragán, Jose Vicente Gil, Mario Rodenas-Rovira, Juan Eduardo Megías-Vericat, Jorge Labrador, José Esteban Peris-Ribera, Jose Luis Poveda-Andrés, Pau Montesinos

**Affiliations:** 1 Pharmacy Department, Hospital Universitari i Politècnic La Fe, Valencia, Spain; 2 Accredited Research Group On Hematology, Instituto de Investigación Sanitaria La Fe (IISLAFE), Valencia, Spain; 3 Accredited Research Group On Pharmacy, Instituto de Investigación Sanitaria La Fe (IISLAFE), Valencia, Spain; 4 Faculty of Pharmacy, Universitat de València, Valencia, Spain; 5 Pharmacy Department. Hospital Reina Sofia, Murcia, Spain; 6 Clinical Pharmacokinetics and Applied Pharmacotherapy Group, Biomedical Research Institute of Murcia (IMIB-Pascual Parrilla), Murcia, Spain; 7 Hematology Department, Hospital Universitari I Politècnic La Fe, Valencia, Spain; 8 Analytical Unit Platform, Instituto de Investigación Sanitaria La Fe (IISLAFE), Valencia, Spain; 9 Molecular Biology Unit, Hospital Universitari i Politècnic La Fe, Valencia, Spain; 10 Research Unit, Hospital Universitario de Burgos, Burgos, Spain; 11 Hematology Department, Hospital Universitario de Burgos, Burgos, Spain

**Keywords:** Acute myeloid leukemia, Population pharmacokinetics, Quizartinib, Newly diagnosed, FLT3-ITD negative, UPLC-MS/MS

## Abstract

**Purpose:**

Quizartinib pharmacokinetics in FLT3-ITD negative acute myeloid leukemia (AML) remain largely unexplored. This study aims to validate a population pharmacokinetics model (popPK) for quizartinib in plasma samples of FLT3-ITD negative AML patients. To do so, an ultra-performance liquid chromatography coupled with tandem mass spectrometry (UPLC-MS/MS) method has been developed and validated for the quantification of quizartinib.

**Methods:**

Plasma samples were collected from FLT3-ITD negative newly diagnosed AML patients undergoing quizartinib therapy at induction in the QUIWI phase II clinical trial [NCT04107727, PETHEMA group] between March 2020 and February 2022. The UPLC-MS/MS method was developed and validated. A previously described popPK model was validated using external validation techniques and implemented using the software NONMEM v7.5.

**Results:**

The developed UPLC-MS/MS method demonstrated high accuracy and precision with a linear range of 6 to 200 ng/mL, with relative standard deviation between 3–11 and accuracy of 88–97% from nominal values. The external validation of the quizartinib popPK model showed minimal bias at the population level (MdPE: -9.86%; ME: 0.50 ng/mL, p = 0.964), but moderate imprecision (MdAPE: 32.28%) and suboptimal accuracy (F20: 24.5%; F30: 43.4%). Individual predictions improved performance, with negligible bias (MdPE: -0.50%), acceptable precision (MdAPE: 11.27%), and F20 (64.2%) and F30 (77.4%) exceeding predefined thresholds. Visual predictive checks confirmed adequate prediction of median concentrations, though some deviations occurred at extremes.

**Conclusion:**

This study presents a replicable UPLC-MS/MS method for the determination of quizartinib in plasma. The validated popPK model can be used to optimize dosing strategies in future clinical studies.

**Supplementary Information:**

The online version contains supplementary material available at 10.1007/s00228-025-03909-4.

## Introduction

Acute myeloid leukemia (AML) is a hematologic malignancy characterized by the uncontrolled proliferation of immature myeloid cells in the bone marrow, leading to impaired normal hematopoiesis and, ultimately, severe marrow dysfunction. Among the various genetic abnormalities involved in AML pathogenesis, mutations in the FLT3 gene are particularly significant. These mutations, present in approximately 25% of AML patients [1], drive aberrant signaling that promotes leukemic cell survival and proliferation and are associated with poor prognosis. In this context, the second-generation selective FLT3 inhibitor, quizartinib, has shown substantial efficacy in FLT3-mutated AML patients, yielding clinical responses by specifically targeting this pathway [2].

However, the potential of quizartinib in patients with *FLT3-ITD* negative AML remains largely unexplored, but recently, studies have begun to analyze its potential effectiveness in this subgroup of patients, as QUIWI trial [NCT04107727] from the PETHEMA group [3]. The lack of pharmacokinetic (PK) data on quizartinib in this population represents a key limitation, as the drug's behavior may differ considerably due to the absence of ITD mutation. This gap in knowledge hinders dose optimization and an understanding of potential side effects in *FLT3-ITD* negative patients, thereby affecting clinical decision-making for this AML subgroup. The development of reproducible and reliable analytical techniques is essential for monitoring plasma levels of quizartinib and generating robust PK data that can inform predictive and personalized models, as in other small molecular inhibitors (SMI) [4].

Research on the development of analytical methods for quantifying quizartinib has focused on liquid chromatography coupled with tandem mass spectrometry (LC–MS/MS), valued for its high sensitivity and specificity. These methods enable precise quantification at nanomolar concentrations, making them crucial for pharmacokinetic studies and therapeutic drug monitoring. Retmana et al. developed and validated a LC–MS/MS method for quizartinib in mouse plasma, employing salt-assisted liquid–liquid extraction (LLE) for sample preparation [5]. Similarly, Ezzeldin et al. validated an LC–MS/MS method for quizartinib quantification in rat plasma, using LLE with dichloromethane as the extraction solvent [6].

Despite these advances, there are limited studies detailing and validating methods for determining quizartinib in human plasma. Sanga et al. investigated the absorption, metabolism, and excretion of quizartinib using radio‐detector high-performance liquid chromatography (radio‐HPLC) and LC–MS after administering a single oral dose of 60 mg of [14C]-quizartinib to six healthy men [7]. This study revealed extensive metabolism, identifying AC886 as the major circulating metabolite, with maximum blood concentration reaching four hours post-dosing and predominant elimination via feces. Li et al. quantified quizartinib and AC886 in human plasma to support dose adjustment strategies [8], while Kang et al. conducted a comprehensive population PK (popPK) analysis of quizartinib in 649 individuals, including healthy volunteers and patients [9].

Additionally, literature on the analysis of other tyrosine kinase inhibitors offers parallels in methodology. For example, Ni et al. and Ezzeldin et al. developed and validated LC–MS/MS methods for analyzing a group of tyrosine kinase inhibitors using LLE with a mixture of ethyl acetate and tert-butyl methyl ether as solvents [10, 11]. Interestingly, quizartinib itself was used as an internal standard (IS) in this and other works, demonstrating the versatility of these approaches and their relevance to the current work.

In this study, we present the validation of a popPK model aimed at characterizing the drug’s distribution in *FLT3-ITD* negative AML patients, as well as the development and validation of a quizartinib analytical method using LLE as sample treatment prior to ultra-performance LC–MS/MS (UPLC-MS/MS). This research aims to provide a dataset to optimize quizartinib administration in this population and to explore its therapeutic potential in this subgroup of patients.

## Materials and methods

To achieve the objective, we conducted a prospective, low-intervention study at a single tertiary hospital. Adult patients (≥ 18 years) newly diagnosed with *FLT3-ITD* negative AML were enrolled. They received a 3 + 7 induction regimen (idarubicin plus cytarabine) combined with quizartinib as part of the QUIWI phase II clinical trial [NCT04107727] between March 2020 and February 2022. The induction scheme involved intensive chemotherapy from day + 1 to day + 7, followed by quizartinib monotherapy from day + 8 to day + 21. This study was approved by the local Ethical Research Committee.

### Chemicals

Quizartinib (> 99% purity) was purchased from Cymit Quimica (Spain) while [^2^H_4_]-Quizartinib (> 95% purity) was purchased from Alsa Chim (France). Ethyl acetate and tert butyl methyl ether, both HPLC grade (99.9%), ACS regent grade acetic acid (99.7%) and ammonium acetate LC–MS grade were all purchased from Merck (Germany). LC/MS grade acetonitrile was purchased from Fisher Scientific (UK).

### Samples

Plasma samples were collected from patients at steady state (two weeks after quizartinib initiation) at predose, + 2-h, + 4-h, and + 6-h. Samples were collected in EDTA tubes with a collection window of ± 15 min, and any incidents related to sampling were recorded. Samples were centrifuged at 3000 g for 5 min at 4 °C, and then the plasma was transferred into a new tube and stored at −80 °C until analysis avoiding freeze–thaw cycles. All samples were thawed and treated on the same day. Due to the trial's double-blind design, samples were also collected from patients not receiving quizartinib to maintain blinding; these were subsequently used as blank samples in the development of the analytical method.

### Stock solution, calibration standards, and quality control samples

Two stock solutions were prepared, one of quizartinib, and another of the IS, [^2^H_4_]-quizartinib. Both were prepared at 20 μg/mL in acetonitrile (ACN) and aliquoted at −20ºC until further use, avoiding repeating freezing–thawing processes. Calibration standards, with concentrations in the 6–200 ng/mL range of the analytes and 80 ng/mL of the IS, were prepared by dilution of these stock solution in initial conditions of mobile phase to obtain calibration standards at concentrations 6, 12, 18.75, 25, 37.5, 50, 75, 100, 150, and 200 ng/mL.

In addition, blank samples, from patients not receiving quizartinib, were spiked with known amounts of the analyte at 20, 80 and 140 ng/mL (categorized as low, middle, and high respectively) and used as quality control (QC) samples. Calibration standards and QC samples were freshly prepared on the day of analysis, never stocked.

### UPLC-MS/MS method

#### UPLC-MS/MS system and conditions

The UPLC-MS/MS system consisted of an Acquity UPLC System and a Xevo TQ-S mass spectrometer (Waters) in the multiple reaction monitoring (MRM) mode with positive electrospray ionization. The analytical column was an Acquity UPLC BEH C18 1.7 μm (2.1 × 100 mm). Mobile phase A consisted of 10 mM ammonium acetate and 0.1% acetic acid in water and B of 0.1% of acetic acid in ACN.

The gradient, at 45 °C and a flow rate of 0.4 mL/min, was: 0–0.2 min, 30% phase B; 0.2–3.0 min linear gradient from 30 to 99%, held for 1 min; 4–4.2 min linear gradient to 30% and held for 1.3 min, thus providing a run time of 5.5 min. The injection volume was 3 μL and the autosampler temperature was set at 6 °C. A capillary voltage of 1 kV, a source temperature of 150 °C and a desolvation temperature of 500ºC were used. Desolvation and the cone gas flow were set as 1000 L/h and 150 L/h, respectively, and the collision gas was 0.17 mL/min. MRM parameters and retention times for quizartinib, and the IS used, isotopically labelled [^2^H_4_]-quizartinib, are summarized in Table 1. Figure S1. in Supplementary Material shows structures of quizartinib and its deuterated internal standard ([^2^H₄]-quizartinib), and their two main fragments.

**Table 1:** Multiple reaction monitoring parameters

Compound	ESI	Compound Precursor	Product ion (quan)	Product ion (qual)	Cone voltage (V)	Collision energy (eV)	Retention time (min)
Quizartinib	+	561.2	420.9	114	40	30/40	2.1
[^2^H_4_]-quizartinib (IS)	+	565.2	118	424.9	40	40/30	2.1

*ESI* Electrospray ionization; *eV* Electronvolt; *IS* Internal Standard; *min* minutes; *V* Volt.

#### Sample preparation

Sample treatment was performed to 100 μL of each plasma sample. First, 30 μL of the IS working solution (480 ng/mL) was added and then a LLE was performed. To do so, 500 μL of a mixture of ethyl acetate and tert butyl methyl ether (1:1) was added to each tube, vortex mixed for 1 min and centrifuged at 10,000 × g for 5 min. Then, 400 μL of the upper organic layer was transferred to a clean tube and dried in a Savant speedvac concentrator (Thermo Electron Corporation). The dried extracts were reconstituted with 145 μL of mobile phase, (70:30 water:ACN, 0.1% v/v acetic acid and 10 mM ammonium acetate), centrifuged and the clear supernatants transferred into 96-well plates for its analysis.

### Method validation

The analytical method in this study was partially validated following the guidelines set by the US FDA and ICH for the validation of bioanalytical methods [12]. The validation process assessed selectivity, linearity, accuracy, precision, matrix effect and recovery. These validation parameters were established to ensure that reliable data was used to build the pharmacokinetic model.

### External validation of population model of quizartinib

#### External evaluation dataset

The external evaluation dataset was sourced from newly diagnosed *FLT3-ITD* negative AML patients of representing clinical conditions, and it was utilized exclusively to evaluate the predictive performance of the quizartinib popPK model in an independent patient cohort.

#### External validation procedure

The quizartinib popPK model was independently implemented in NONMEM version 7.5 (Icon Development Solutions, Ellicott City, MD, USA) [9]. Parameter values and covariate relationships were fixed according to those reported in the original publication [9], without any re-estimation or modification. Key model parameters were described at Table 2:

**Table 2:** Summary of pharmacokinetic parameters and drug concentration predictions

	**Mean**	**SD**	**Median**
CL	1.97	0.56	1.76
KE	0.01	0.00	0.01
KA	1.68	0.01	1.68
F1	0.77	0.23	0.75
t_1/2_	82.38	22.76	82.50
V2	219.28	28.18	227.32
V3	174.39	35.84	176.03
Q3	26.13	2.60	26.56
Q4	0.56	0.00	0.56
KA	1.68	0.01	1.68
V4	39.30	0.00	39.30
DV	129.08	57.86	135.00
IPRED	126.59	46.47	131.04
PRED	121.79	16.78	124.90
NPDE	0.12	0.95	0.31

CL Clearance; KE Elimination rate constant; KA Absorption rate constant; F1 Bioavailability; t_1/2_Half-life; V2 Volume of distribution (central compartment); V3 Volume of distribution (peripheral compartment); Q3 Inter-compartmental clearance; Q4 Inter-compartmental clearance; V4 Volume of distribution; DV Drug concentration at time t; IPRED Individual predicted concentration; PRED Population predicted concentration; NPDE Normalized Prediction Distribution Error.

Predictions were generated using recorded doses, sampling times, and covariate values from the evaluation dataset.

#### Evaluation of predictive performance

Goodness-of-fit plots compared population and individual predictions with observed concentrations, visually assessing precision and bias [13, 14]. The correlation coefficient (R^2^) quantified the relationship between observed and predicted concentrations for both population and individual predictions.

Model performance was assessed through statistical methods to evaluate bias, inaccuracy, and predictive accuracy. Metrics used included mean prediction error (MPE, Eq. 1), relative prediction error (PE, Eq. 3), and absolute prediction error (APE, Eq. 4), calculated as follows:

1 $$\text{MPE }\left(\text{ng}/\text{mL}\right)=Cpred-Cobs$$

2 $$\mathrm{PE}\;(\%)=\frac{Cpred-Cobs}{Cobs}\times100$$

3 $$\mathrm{APE}\;\left(\%\right)=\left|\mathrm{PE}\right|\times100$$

where *Cpred* and *Cobs* represent predicted and observed concentrations, respectively. Median prediction error (MdPE, Eq. 4) and median absolute prediction error (MdAPE), Eq. 5) were calculated to assess bias and inaccuracy, respectively:

4 $$\mathrm{MdPE}\;\left(\%\right)=\mathrm{MEDIAN}\left(\mathrm{PE}\left(\%\right)\right)$$

5 $$\mathrm{MdAPE}\;\left(\%\right)=\mathrm{MEDIAN}\left(\mathrm{APE}\left(\%\right)\right)$$

Acceptability criteria were defined as MdPE within −20% to 20% and MdAPE ≤ 30%, and F20 ≥ 35%, and F30 ≥ 50% [15].

#### Simulation-based Diagnostics

The predictive performance of the model was evaluated using simulation-based diagnostic methods, specifically through prediction-corrected visual predictive check (pcVPC) implemented in an R package. The pcVPC plots were generated to compare the prediction-corrected concentrations in the external cohort. In this analysis, the median, 5th, and 95th percentiles of the prediction-corrected concentrations were compared with the 95% confidence intervals for these percentiles generated from 1000 simulated datasets, assessing the agreement between both distributions. pcVPCs allow the comparison of the distribution of observations and predictions against an independent variable, time in this case.

### Statistical analysis

The trapezoidal rule was used to calculate the Area Under Curve (AUC), due to samples were at steady state. Quantitative variables were expressed as mean and standard deviation (SD). Categorical variables were shown with frequency and percentage. Statistical analysis was performed using Stata 14.2 software.

## Results

The dataset consisted of newly diagnosed wild-type FLT3 AML patients treated with quizartinib (Table 3). Each patient took one 30 mg film-coated tablet of quizartinib orally of quizartinib daily with food, except one, who received 60 mg. This dose reduction was necessary due to concomitant administration of voriconazole (200 mg every 12 h), a strong cytochrome P450 (CYP) 3A4 inhibitor.

**Table 3:** Baseline characteristics of the population (n = 14)

Characteristic (units)	Number (%)	Mean (SD)
Female	9 (64)	
Age (years)		54 (13)
ECOG (number)		0.6 (0.8)
WBC (× 10^9^/L)		26 (49)
Hemoglobin (g/dL)		9 (2)
Quizartinib with food	14 (100)	
Strong CYP3A4 inhibitor	11 (79)	

*CYP3A4* cytochrome P450 3A4; *ECOG* Eastern Cooperative Oncology Group Performance Status Scale; *SD* Standard Deviation; *WBC* White Blood Count.

A total of 53 plasma samples extracted at steady state were analyzed from 14 patients receiving quizartinib. Thirteen patients provided a complete set of samples, while one patient provided only the + 6-h sample.

### Analytical method validation

#### Calibration curve

The quizartinib calibration standards were prepared by serial dilution of the highest calibration solution in initial conditions of mobile phase, with concentrations in the 6–200 ng/mL range of the analyte and 80 ng/mL of the IS. This range was enough to quantify all the samples in the study.

The area peak ratios between quizartinib and the IS were plotted against quizartinib concentration using a weighted linear least square regression, obtaining correlation coefficients (r^2^) of 0.9902 (y = 0.0116x + 0.1429).

#### Selectivity

To study selectivity, six different blank plasma samples (from patients who had not taken the medication) were analyzed to study the presence of interferences in the region in which the elution of each analyte is expected. In all cases there was no interference, and the signal was less than 2% of that of the limit of quantification.

**Fig. 1:**
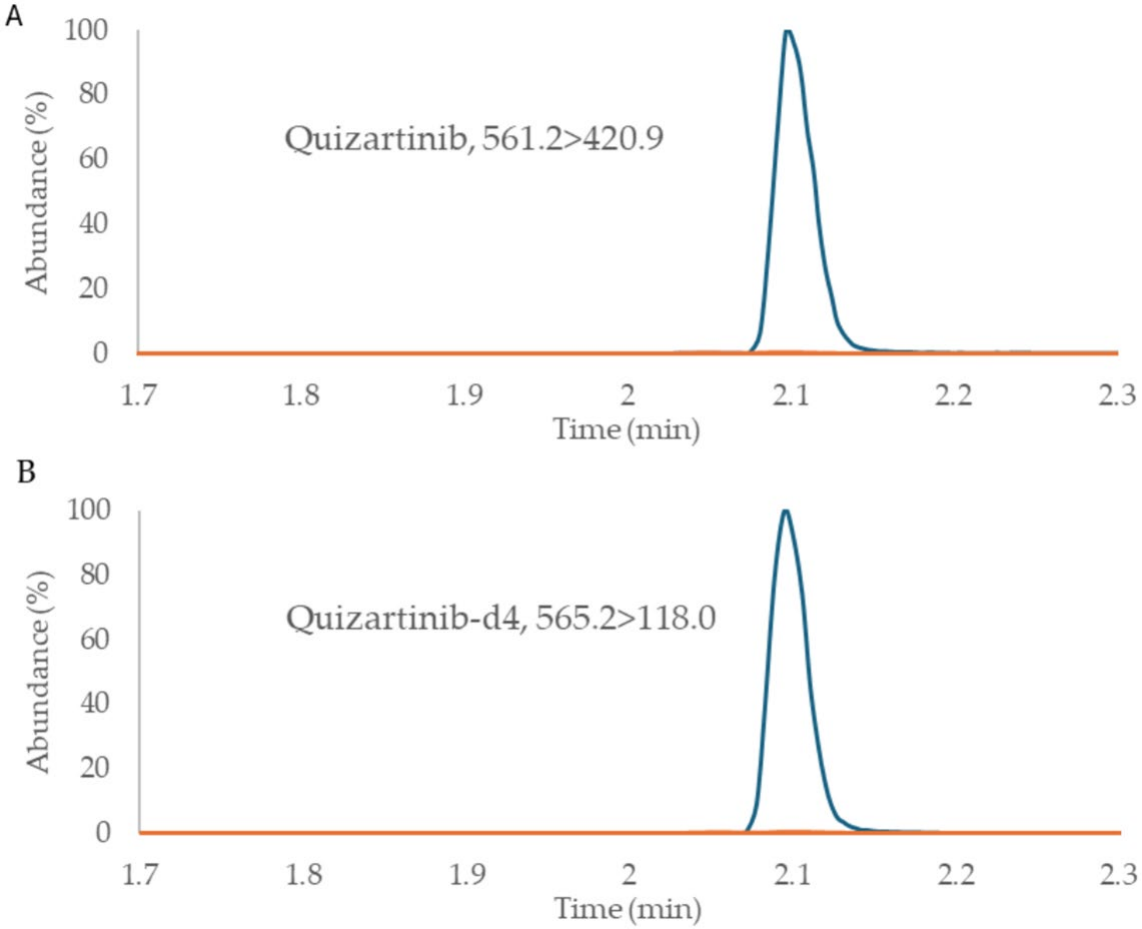
Representation MRM chromatograms of quizartinib (**A**) and internal standard (**B**) in a representative sample (in blue) compared to a blank sample (in orange)

A representative MRM chromatogram of quizartinib and [^2^H_4_]-quizartinib in a batch sample and a blank plasma is shown in Fig. 1.

#### Carry over

The carryover was determined using a blank injected just after the standard at the highest concentration level. In all cases the signal obtained in the region was lower than 20% of that of the limit of quantification.

#### Sensitivity

The lowest concentration on the calibration curve, 6 ng/mL, was designated as the LLOQ (Lower Limit of Quantification) for the method. This level met the required criteria of a signal-to-noise ratio of at least 10, accuracy within 80–120% of the nominal values, and relative standard deviation (RSD) lower than 20%. While this sensitivity could potentially be improved further, this was found unnecessary, as the established LLOQ was sufficient to quantify all plasma samples in the study.

#### Accuracy and precision

The QC samples (blank samples spiked at high, intermediate and low concentration) were used to assess the precision and accuracy of the method. Precision and accuracy were determined by performing six replicates of each QC level on three separate days (18 QC samples per day). RSD were calculated for both the intra and inter day to study precision while the mean percentage deviation from the corresponding nominal value was calculated to study accuracy.

Very good values were obtained, with an intra-day precision ranged between 3 and 8% and inter-day between 7 and 11%. Also, accuracy ranged between 88–97%. Table 4 summarizes precision and accuracy data.

**Table 4:** Intra‐day and inter‐day precision and accuracy of quizartinib

Nominal concentration (ng/mL)	Intra-day				Inter-day		
	Mean ± SD (ng/mL)	Precision (RSD, %)	Accuracy (%)		Mean ± SD (ng/mL)	Precision (RSD, %)	Accuracy (%)
20	17.7 ± 1.5	8.5	88		19 ± 2	11	96
80	77 ± 4	5	96		78 ± 6	8	97
140	127 ± 4	3	91		129 ± 7	7	92

*SD* Standard Deviation; *RSD* relative standard deviation.

#### Matrix effect and extraction recovery

The matrix effect of the method was evaluated at low, medium, and high QC levels using blank plasma from three different individual donors. For each matrix and level, three replicates were prepared by extracting blank plasma samples, followed by post-extraction spiking of analyte and internal standard. Signals were compared to equivalent concentrations of standard solutions. The analyte and internal standard peak areas were both reduced by the same proportion, and the average ratio of peak area in the post-extraction spiked samples to that in standard solutions was 0.70, indicating a 30% ion suppression. The RSD (%) of this matrix effect was below 10%, demonstrating acceptable consistency across matrices. Despite this suppression, no IS-normalized matrix effect was observed, as the response factor (analyte-to-IS peak area ratio) remained constant, ensuring reliable quantification and supporting the good accuracy obtained.

Similarly, recovery was evaluated by comparing pre-extraction and post-extraction spiked samples at low, medium, and high QC levels. Both analyte and internal standard peak areas were reduced by approximately 50% in pre-extraction samples, indicating a recovery of 50% for the liquid–liquid extraction. The RSD was below 15% in all cases. Nevertheless, the IS-normalized recovery was close to 100%, as the internal standard effectively corrected for extraction losses, maintaining accuracy and reproducibility of the method.

Overall, the internal standard, due to its closely matched chemical and physical properties, effectively compensated for both matrix effects and extraction losses, ensuring robustness of the method.3.2.

### Validation dataset description

Mean concentrations at predose, + 2-h, + 4-h and + 6-h were 82 (41), 129 (58), 141 (59) and 120 (53) ng/mL and AUC varied from 718 to 4372 ng/mL·h.

The main PK parameters estimated for quizartinib showed variability across the dataset. The median clearance (CL) was 1.76 L/h, while the absorption rate constant (KA) had a median value of 1.67 h^ − 1^. The bioavailability factor (F1) had a median of 0.75. For the volume of distribution, median values were as follows: V2 (central compartment) at 212.9 L, V3 (peripheral compartment) at 176.03 L, and V4 at 39.30 L. Intercompartmental clearances were represented by Q3 at 26.36 L/h and Q4 at 0.56 L/h.

#### Comparison of observations vs. population predictions

The external evaluation of the quizartinib popPK model indicated that the model partially met the predefined acceptability criteria. The MdPE was −9.86%, which fell within the acceptable range of −20% to 20%, suggesting minimal systematic bias. The MPE was 0.50 ng/mL (SD:80.73; p = 0.964) further supporting the absence of statistically significant overall bias. However, the MdAPE was 33.28% exceeding the predefined threshold of 30%, indicating moderate imprecision in individual predictions. Furthermore, the proportions of predictions within ± 20% (F20: 24.5% and ± 30% (F30: 43.4%) of observed concentrations failed to meet the required thresholds of ≥ 35% and ≥ 50%, respectively, underscoring limitations in predictive accuracy at the individual level. Prediction errors were evaluated by stratifying observations into low (< 100 ng/mL), medium (100–150 ng/mL), and high (> 150 ng/mL) concentration ranges. The MdPE was + 43.5% at low, –0.66% at medium, and –12.2% at high concentrations.

#### Comparison of observations vs. individual predictions

A substantial improvement in predictive performance was observed when individual predictions were evaluated instead of population predictions. The MPE was 0.937 ng/mL (SD: 25.29;p = 0.788) indicating no statistically significant overall bias in the individual-level predictions. The MdPE was 0.50%, reflecting negligible systematic bias, while the MdAPE was 11.27%, demonstrating acceptable precision within individual predictions. Furthermore, the proportion of individual predictions within ± 20% (F20) and ± 30% (F30) of observed concentrations improved markedly to 64.2%and 77.4%, respectively, exceeding the predefined acceptability thresholds (F20 ≥ 35% and F30 ≥ 50%).

#### Goodness-of-Fit and Residual Analysis

**Fig. 2:**
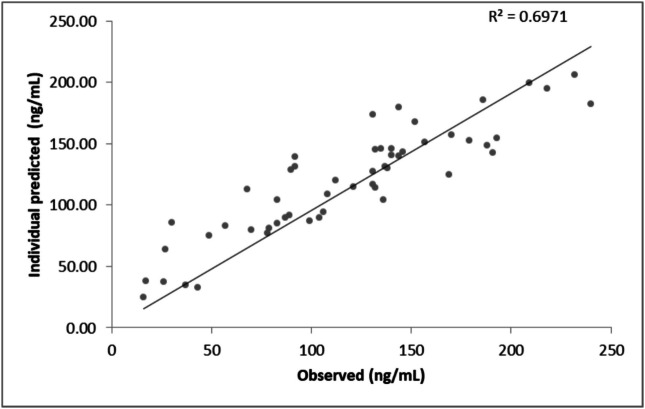
Individual predictions concentrations versus observed concentrations (ng/mL)

Goodness-of-fit analysis demonstrated moderate predictive performance of the popPK model, with a coefficient of determination (R^2^) of 69.71%, indicating that the model explained a substantial proportion of the observed variability (Fig. 2). However, some degree of unexplained variability persisted, particularly at higher concentration levels. Visual inspection of the observed versus individual predicted concentrations revealed a general alignment along the line of identity, although certain deviations were noted at the extremes.

#### Diagnostics Based on Simulations

**Fig. 3:**
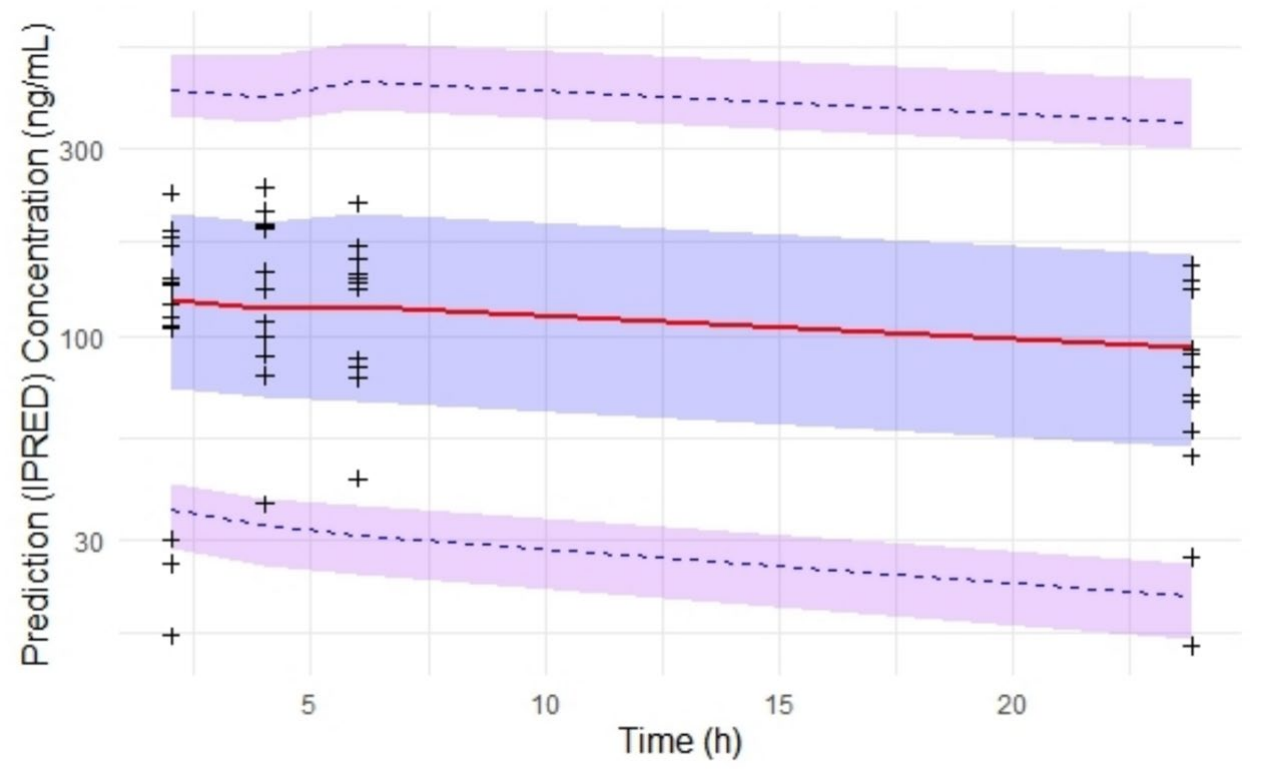
Prediction-Corrected Visual Predictive Check (pcVPC) for the pharmacokinetic model of Quizartinib using individual predicted concentrations (IPRED). The red line represents the observed median concentration over time. The blue shaded area indicates the 90% prediction interval (PI) for the predicted concentrations, while the purple shaded areas represent the 5th and 95th percentiles of the observed data

The pcVPC plot (Fig. 3) provided a visual assessment of the model’s predictive performance over time. The observed median concentrations closely aligned with the model-predicted median, and most observed data points were contained within the 90% prediction interval. This suggests that the model adequately captured both the central tendency and variability in quizartinib concentrations across the population. Minor discrepancies were observed at the extremes, indicating that the model may not fully account for variability at the upper and lower concentration ranges.

## Discussion

The advent of targeted therapies has revolutionized the treatment landscape for AML, bringing forth new challenges distinct from those posed by conventional chemotherapy. A key challenge is the transition from intravenous to oral administration, which heightens the risk of drug-drug interactions and increases variability in plasma concentrations. This shift underscores the critical need to monitor plasma levels, aiming to determine whether they can reliably predict both therapeutic efficacy and adverse effects associated with SMI.

Quizartinib, initially approved for treating relapsed FLT3-mutated patients, is now under investigation for potential use in *FLT3-ITD* negative AML patients, spurred by emerging insights into distinct biological mechanisms [16]. Expanding its use in this population presents added complexities, as these patients may exhibit variability in factors such as FLT3 ligand levels, FLT3 receptor expression, and plasma drug concentration [17, 18]. These variables are believed to significantly influence treatment efficacy, highlighting the importance of developing and validating PK techniques and models that allow us to optimize the treatment individually based on the characteristics of each patient to guarantee clinical results with the greatest safety, as in this study.

Although several studies have investigated the determination of quizartinib using chromatographic techniques, the majority have been conducted in animal models or healthy subjects[6, 7, 9], limiting the extrapolation of their findings to clinical patient samples. Furthermore, pharmacokinetic studies frequently lack detailed descriptions of the analytical methodologies employed, thereby hindering the reproducibility of these analyses by other researchers. To address these limitations, the present study incorporates a detailed description of the analytical technique alongside the validation of the pharmacokinetic model, providing a novel focus.

This study represents the first to evaluate quizartinib concentrations and external validation of a pharmacokinetic model in *FLT3-ITD* negative AML patients. Notably, a recent retrospective analysis by Vaddady et al. examined quizartinib concentrations in Phase I, II, and III clinical trials, confirming the impact of CYP3A4 inhibitors on quizartinib exposure and identifying time-dependent variations in quizartinib concentrations, with higher levels observed during the consolidation phase [19]. Future investigations incorporating our validation within patient samples from the consolidation schemes would be of significant value to further assess the model's applicability. Additionally, Vaddady et al. demonstrated the concentration-dependent effect of quizartinib on QT interval prolongation [20], underscoring the imperative for therapeutic drug monitoring due to the heightened risk of cardiovascular events associated with quizartinib in AML patients[21]. These findings highlight the critical importance of individualized pharmacokinetic evaluation to optimize safety and efficacy in this clinical context.

The application of popPK models in clinical practice requires robust external validation to ensure predictive performance. To our knowledge, this is the first study to conduct an external validation of a popPK model for quizartinib. The evaluation in FLT3-ITD-negative AML patients showed that the model partially fulfilled predefined acceptability criteria. At the population level, the model demonstrated minimal bias (MdPE: −9.86%; MPE: 0.5 ng/mL, p = 0. 9641), but exhibited moderate imprecision (MdAPE: 33.28%) and suboptimal accuracy (F20: 24.5%; F30: 43.4%). These findings suggest that while the model captured central tendencies well, its precision and individual-level predictive accuracy were limited. In contrast, individual predictions (IPRED) showed substantial improvement, with negligible bias (MPE: 0.937 ng/mL, p = 0.788; MdPE: −0.5%), improved precision (MdAPE: 11.27%), and F20 and F30 values exceeding acceptability thresholds (64.2% and 77.4%, respectively). The pcVPC confirmed that the model adequately described the median and variability of quizartinib concentrations, though minor deviations at extremes suggest residual variability. Overall, this model is suitable for individual predictions and dose optimization, but further refinement may improve population-level performance, particularly in patients with extreme concentrations. The evaluation of residuals across the full concentration range revealed moderate bias at the extremes, with a tendency to overpredict at low concentrations and underpredict at high concentrations. Although the model demonstrated good predictive performance in the medium concentration range. Future efforts should explore additional covariates to enhance predictive precision, as genetic polymorphisms.

A limitation of this research is the lack of monitoring for the active metabolite AC886, which is incorporated in Kang's model [9]. Although this determination was attempted, we decided not to include the data in the model for different reasons: 1) The validation of the analytical method for this compound was unsatisfactory. The IS ([^2^H_4_]-quizartinib) did not adequately correct the metabolite signal, resulting in poor precision and accuracy when using IS calibration. Switching to external calibration significantly improved precision; however, accuracy remained around 50%, constant at all three levels, probably due to the efficiency of the LLE. This issue could likely be resolved by adjusting the treatment to better suit the metabolite determination or by finding an IS that works effectively in this case, ideally the isotopically labeled version of the metabolite itself, which, as far as we know, is not commercially available. Another solution would be to correct the data obtained considering this accuracy, as it was not near 100%, but the 50% accuracy obtained seemed consistent, precise, and reproducible. 2) To enable a simplified adjustment method that excludes the active metabolite, thus allowing for easier dose adaptation. While the metabolite may indeed influence treatment effectiveness, in this case, simplifying the model as much as possible would be ideal to facilitate the integration of Therapeutic Drug Monitoring (TDM) into routine clinical practice. 3) The sparse sampling during the elimination phase limits the model’s capacity to accurately characterize the drug’s elimination kinetics. This data limitation may compromise the precision of the VPCs plots, as the prediction intervals might not fully capture the variability and actual behavior of concentrations in the terminal phase.

Another limitation of our study is the low number of patients included for external model validation of popPK model, which may impact the robustness of our conclusions. Even so, taking four samples at different times from each patient allows for obtaining enough samples to conduct the popPK validation. It should also be emphasized that despite many hospital services do not have equipment like UPLC-MS/MS, one option would be to centralize these TDM analyses in reference centers, which would enhance efficiency. Moreover, despite overestimating some results, the popPK model is reliable and could enable future studies on quizartinib toxicities and efficacy, paving the way for personalized treatments with TDM.

## Conclusions

In this work, a popPK model was validated, allowing prediction of quizartinib concentrations in *FLT3-ITD* negative AML patients and enabling a detailed characterization of its PK parameters. Additionally, a UPLC–MS/MS method for determining quizartinib was developed and validated. The proposed chromatography procedure is simple and selective for the extraction of the analyte, and the popPK model is robust and has the potential to support future research on the toxicities and efficacy of quizartinib, which could further refine its therapeutic monitoring and improve outcomes for this patient population.

## Supplementary Information

Below is the link to the electronic supplementary material.

Supplementary file1 (DOCX 486 KB)

## Data Availability

The data that supports the findings of this study are available from the corresponding author, AS-A, upon reasonable request, due to privacy or ethical restrictions.

## Declarations

Institutional Review Board Statement: The study was conducted in accordance with the Declaration of Helsinki and approved by the Institutional Review Board (or Ethics Committee) of Instituto de Investigación Sanitaria La Fe (IISLAFE) of Valencia (protocol code 2020–017-1, and date of approval 11th March 2020).

Informed Consent Statement: Informed consent was obtained from all subjects involved in the study.
