# Supplementary material for: Validation of pharmacokinetic model for quizartinib quantified by UPLC-MS/MS in patients with FLT3-ITD negative newly diagnosed acute myeloid leukemia
Source: Eur J Clin Pharmacol. 2025 Aug 30;81(11):1699–709. doi: 10.1007/s00228-025-03909-4 (PMC12511262; doi:10.1007/s00228-025-03909-4)

**Figure S1.** Structures of quizartinib and its deuterated internal standard ([²H₄]-quizartinib), showing the main fragments used for quantification.


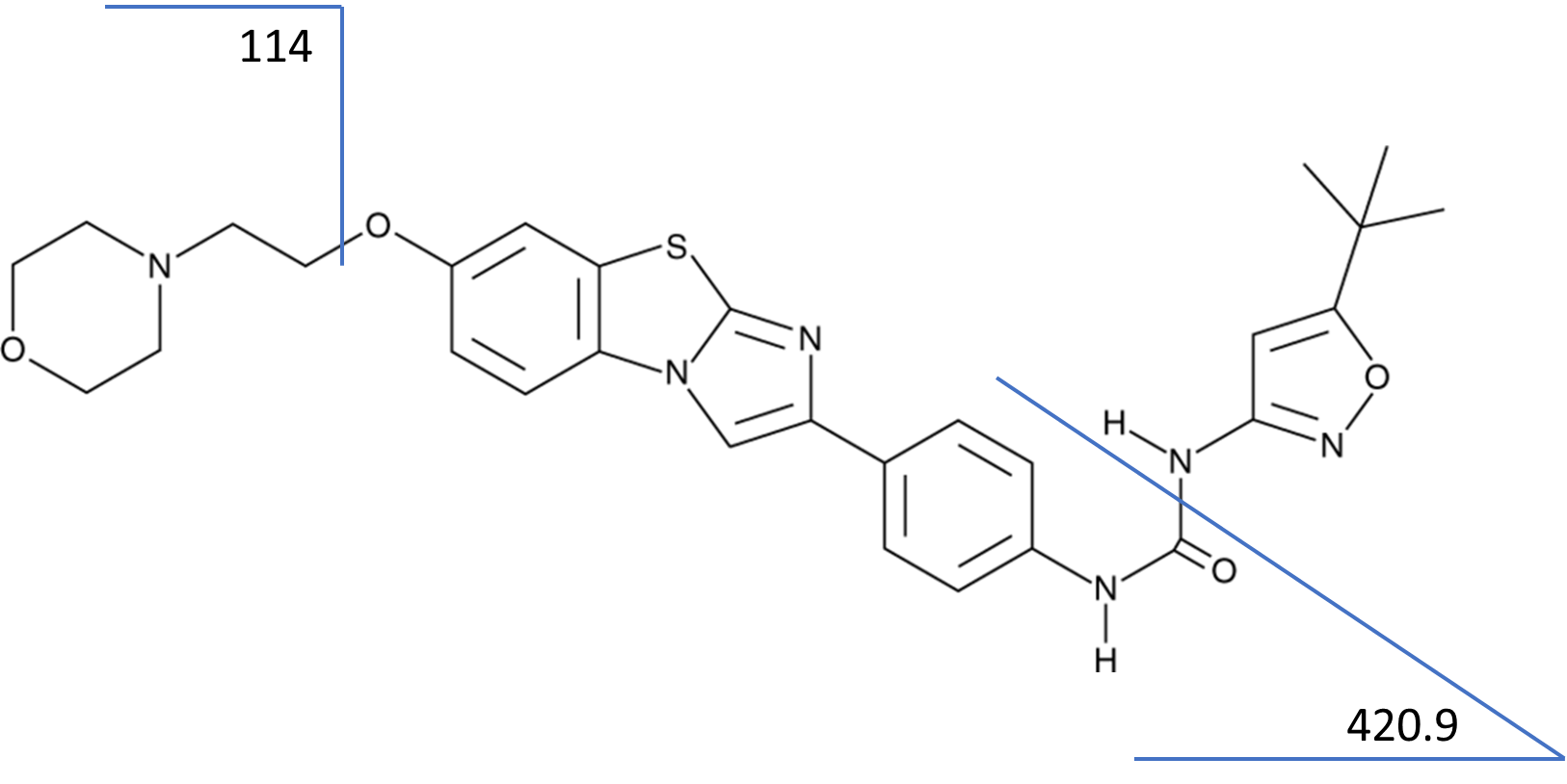

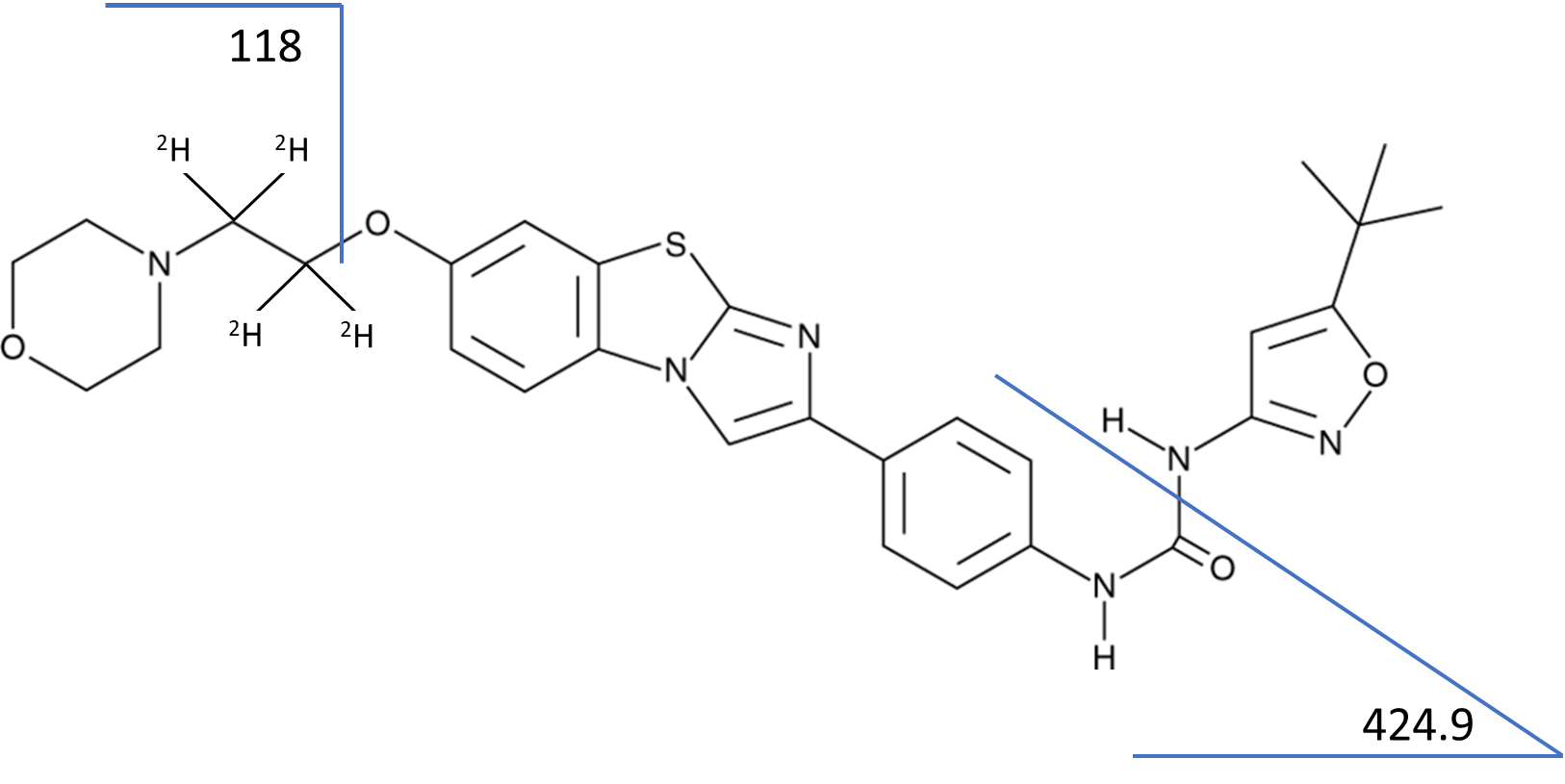

Supplement: Supplementary file 1 — Supplementary file1 (DOCX 486 KB) [file 228_2025_3909_MOESM1_ESM.docx]
